# The role of Savings and Internal Lending Communities (SILCs) in improving community-level household wealth, financial preparedness for birth, and utilization of reproductive health services in rural Zambia: a secondary analysis

**DOI:** 10.1186/s12889-022-14121-9

**Published:** 2022-09-12

**Authors:** Ha Eun Lee, Philip T. Veliz, Elisa M. Maffioli, Michelle L. Munro-Kramer, Isaac Sakala, Nchimunya M. Chiboola, Thandiwe Ngoma, Jeanette L. Kaiser, Peter C. Rockers, Nancy A. Scott, Jody R. Lori

**Affiliations:** 1 Center for Global Health Equity, University of Michigan, 2800 Plymouth Rd, 100 NCRC, Ann Arbor, MI 48109-5482 USA; 2 Applied Biostatistics Laboratory, University of Michigan, School of Nursing, 400 N. Ingalls St., Ann Arbor, MI 48109-5482 USA; 3 Health Management and Policy, University of Michigan, School of Public Health, 1415 Washington Heights, Ann Arbor, MI 48109 USA; 4 Health Behavior and Biological Sciences, University of Michigan, School of Nursing, 400 N. Ingalls St., Ann Arbor, MI 48109-5482 USA; 5 Africare Zambia, Flat A, Plot 2407/10 MBX, Off Twin Palm Road, Ibex Hill, Box 33921, Lusaka, Zambia; 6 Department of Research, Right to Care Zambia, Ground Floor, Mikwala House, Plot No 11059, Off Brentwood Lane, Longacres, Lusaka, Zambia; 7 Department of Global Health, Boston University School of Public Health, 715 Albany St., Boston, MA 02118 USA; 8 Global Affairs, University of Michigan, School of Nursing, 400 N. Ingalls St., Ann Arbor, MI 48109-5482 USA

**Keywords:** Access to care, Savings group, Reproductive health, Maternal health

## Abstract

**Background:**

Savings and Internal Lending Communities (SILCs) are a type of informal microfinance mechanism widely adapted in Zambia. The benefits of SILCs paired with other interventions have been studied in many countries. However, limited studies have examined SILCs in the context of maternal health. This study examined the association between having access to SILCs and: 1) household wealth, 2) financial preparedness for birth, and 3) utilization of various reproductive health services (RHSs).

**Methods:**

Secondary analysis was conducted on baseline and endline household survey data collected as part of a Maternity Waiting Home (MWH) intervention trial in 20 rural communities across seven districts of Zambia. Data from 4711 women who gave birth in the previous year (baseline: 2381 endline: 2330) were analyzed. The data were stratified into three community groups (CGs): CG1) communities with neither MWH nor SILC, CG2) communities with only MWH, and CG3) communities with both MWH and SILC. To capture the community level changes with the exposure to SILCs, different women were randomly selected from each of the communities for baseline and endline data, rather than same women being surveyed two times. Interaction effect of CG and timepoint on the outcome variables – household wealth, saving for birth, antenatal care visits, postnatal care visits, MWH utilization, health facility based delivery, and skilled provider assisted delivery – were examined.

**Results:**

Interaction effect of CGs and timepoint were significantly associated only with MWH utilization, health facility delivery, and skilled provider delivery. Compared to women from CG3, women from CG1 had lower odds of utilizing MWHs and delivering at health facility at endline. Additionally, women from CG1 and women from CG2 had lower odds of delivering with a skilled provider compared to women from CG3.

**Conclusion:**

Access to SILCs was associated with increased MWH use and health facility delivery when MWHs were available. Furthermore, access to SILCs was associated with increased skilled provider delivery regardless of the availability of MWH. Future studies should explore the roles of SILCs in improving the continuity of reproductive health services.

**Trial registration:**

NCT02620436.

## Background

Utilization of reproductive health services (RHSs) during pregnancy, childbirth, and the postnatal period are critical to ensure women and their babies reach their full potential for health and well-being [1]. These services include but are not limited to: antenatal care (ANC) visits, postnatal care (PNC) visits, maternity waiting home (MWH) utilization, health facility (HF) delivery, and skilled provider (SP) assisted delivery. Timely access to quality RHSs can prevent most maternal morbidity and mortality [2]. Yet, in 2017, more than 295,000 women died worldwide both during and following pregnancy and childbirth [1]. Approximately 94% of all maternal deaths occur in low and middle-income countries (LMICs) and 68% in sub-Saharan Africa [3]. In these settings, limited financial resources are one of the main causes for delays in seeking, reaching, and receiving RHSs [2].

Access to and utilization of RHSs remain highly inequitable, varying markedly with women’s socioeconomic status [4]. Studies have found strong and consistent evidence that utilization of various RHSs are higher among women with more financial resources [4–6]. For example, a recent systematic review examining the determinants of ANC utilization in sub-Saharan Africa found income and employment as enablers to ANC service utilization in sub-Saharan Africa [7], while another review found higher PNC attendance among women with greater household wealth in LMICs since they can afford the medical, non-medical, and opportunity costs associated with PNC visits [4]. Well-known financial barriers to facility-based and SP assisted delivery more generally persist in LMICs, including transportation costs, informal service fees, and purchase of birth items such as baby blankets and plastic sheets for delivery that the health facility may not provide [8]. Even utilization of MWHs, dwelling places for pregnant women to await delivery aimed at reducing access barriers to facility-based delivery, are often hindered by financial barriers including fees for accommodation, food, and transportation costs [9, 10].

Savings Group (SG) is an umbrella term used to describe informal microfinance mechanisms, such as Savings and Internal Lending Communities (SILCs) [11, 12]. Unlike formal microfinance mechanisms, SGs can begin without much external funding and allow participants to access basic financial services to save and borrow money to generate income or to pay for life events such as pregnancy and childbirth [11–13]. Hence, SGs have been identified as a promising intervention to financially empower individuals and communities in rural areas of LMICs and to further address financial barriers to utilizing RHSs [13]. Through regular member meetings, SGs foster additional in-tangible benefits, including sharing of ideas and stories, and generate a sense of belonging and trust among their members [14]. Studies consistently find that SGs increase social capital, often defined as networks of social interaction that are linked to resource exchange [11, 15].

Because SGs are shown to build trust, solidarity, and collective efficacy, they are often used as a social platform to deliver various health and non-health interventions [16]. For example, SGs have been used as a social platform to deliver maternal and child health educational interventions to their members. However, limited studies examine these groups as a financial mechanism to help overcome the financial barriers to accessing and utilizing RHSs [14, 16]. While there are many different types of SGs that have been developed and facilitated by over 70 organizations worldwide, this study examines SILCs, a SGs model developed by Catholic Relief Services [17, 18]. SILCs is one of the most widely implemented SGs in Zambia [18, 19].

To assess the effect of SILCs on access to and utilization of RHSs, a sub-study was conducted within a larger MWH evaluation in rural Zambia [20, 21]. Zambia, a Southern African country, continues to experience high maternal mortality, with 213 maternal deaths per 100,000 live births [22]. As rural Zambian women have lower rates of facility-based delivery with a SP and have repeatedly cited costs as barriers to accessing RHSs, this provided a prime context to assess the effects of having access to SILCs [20, 21]. This article explores the association between access to SILCs and: 1) household wealth, 2) financial preparedness for birth, and 3) utilization of RHSs (ANC, PNC, MWHs, SP delivery, HF delivery). This study hypothesizes that women from communities that have access to both SILCs and MWHs will have higher household wealth, financial preparedness for birth and utilization of RHSs compared to women form communities with only MWH or neither MWH nor SILCs. While MWHs are not the primary intervention of interest, design of the research allowed examination of both interventions, separately and in tandem.

## Methods

### Study setting

MWHs have existed in Zambia for decades with generally low quality and no specific policy to keep them at a particular standard [20]. The Maternity Home Alliance (MHA), a collaboration of two implementing partners, two academic partners, and the Government of Zambia implemented MWHs using a Core MWH Model with specific standards and policies [20]. The MWH parent study was conducted in seven primarily rural districts: Nyimba, Lundazi, Choma, Kalomo, and Pemba, Mansa and Chembe. Characteristics of these districts as well as the core MWH model figure are thoroughly explained elsewhere (20).

One implementing partner (Africare-Zambia), operating in Lundazi, Mansa, and Chembe districts, also implemented SILCs from the beginning of January 2016, within their MWH intervention sites. By the end of October 2017, there were more than 310 active SILCs with 6711 participants from the 10 different communities with the core MWH model. The core MWH models were implemented between June 2016 and August 2018 [23].

Of the seven districts included in the overarching parent study, Kalomo, Mansa, Nyimba, and Lundazi were part of the first phase of Saving Mothers Giving Life (SMGL) initiative [24]. SMGL is a 5-year initiative that was implemented from 2012 to 2016 as a multi-lateral initiative to reduce maternal and newborn mortality [24]. The SMGL approach included a variety of interventions such as training community health workers responsible for improving the knowledge and access to RHSs within their local communities, and mentoring health facility staff to increase quality of care, improving the referral system, and investing in supply chain and facility equipment [10, 25]. The baseline Household Survey (HHS) data were collected in April and May of 2016, overlapping with the SMGL initiative which ended December of 2016 [24].

### Design

A secondary analysis was conducted on two cross-sectional samples of recently delivered women surveyed at baseline (March to May 2016) and endline (August to September 2018) for the MHA impact evaluation. MWHs aim to improve maternal and neonatal health outcomes for the most rural women, who live far from health services by increasing access to facility-based delivery services with a SP [20]. The MHA evaluated the impact of MWH on RHS access, assessed primarily through delivery at a HF. Both baseline and endline HHS data were collected from the communities surrounding 40 rural health centers in seven rural districts of Zambia. Each community had at least one health center capable of managing basic emergency obstetric and neonatal complications (BEmONC) where the core MWH model was implemented nearby [20]. The MWH core model was implemented in 20 of the communities and the remaining 20 communities were used as a control, with a health facility present but no MWH model implemented. The details of the MWH parent study design and data collection process are described elsewhere [20, 21].

Written informed consent was sought from the original study participants and this study was conducted using the de-identified dataset. Ethical approvals for the MWH project were obtained from the authors Institutional Review Boards (IRBs), as well as from the ERES Converge Research IRB, a private local ethics board in Zambia.

### Participants

The parent study used a multistage random sampling procedure for both baseline and endline HHS data (goal of 2400 women) with a probability for village selection proportionate to population size [20]. A household was defined as a group of people who regularly cook together. HHS data were collected from two cross-sectional samples within the sample villages at baseline and endline. Eligibility criteria for women to participate in the HHS included: 1) delivered a baby within the past 12 months, 2) 15 or older (if aged 15–17, a legal guardian had to consent), and 3) resident of the community identified for sampling. If the women who gave birth was deceased, a proxy participant who is 18 or older, took the HHS [20]. To capture the community level changes, different women from the same community were followed at baseline and endline.

The total sample was separated into three CGs: CG1) communities with neither the core MWH model nor SILC (20 communities), CG2) communities with only the core MWH model (10 communities), and CG3) communities with both the core MWH model and SILC (10 communities). All communities included in the study had a BEmONC health facility.

Of the 2381 participants from baseline HHS, 1031participans were from CG1, 597 participants from CG2, and 756 participants from CG3. Of the 2330 participants from endline, 1113 participants were from CG1, 610 participants from CG2, and 598 participants from CG3.

### Measures

Our primary outcomes of interests are: 1) household wealth, 2) financial preparedness for birth, and 3) utilization of RHSs. Variables for demographics, household wealth, saving for delivery, and utilization of RHSs were extracted from a de-identified HHS dataset.

Demographic variables included women’s age, marital status, number of pregnancies, number of livebirths, and education level.

Household wealth was assessed by using the comprehensive list of wealth indicator variables. A total of 57 dichotomized variables included ownership of household assets and quality of housing and water supply that are similar to the variables used in the Demographic and Health Survey (DHS) [26]. Principal component analysis (PCA) was used to assign weights to each of the wealth indicator variables, summed, and created into quintiles – poorest, poor, middle, rich, and richest [26, 27]. PCA is a data reduction procedure where a set of correlated variables are replaced with a set of uncorrelated variables representing unobserved characteristics of the sample [28]. Therefore, wealth indicator variables that are more unequally distributed across the sample will have higher weight. While PCA has its own limitations, using PCA to develop wealth quintiles is one the most frequently used methods by the World Bank and is used in more than 76 countries [26, 27]. We excluded observations that was missing any of the 57 wealth indicator variables and created the wealth quintiles twice, once for the baseline sample and once for the endline sample. This allowed us to understand the wealth distribution between the CGs at baseline and endline.

Financial preparedness for birth was determined by whether women saved any money for their most recent delivery or not.

Utilization of RHSs was examined by the number of ANC and PNC visits, utilization of MWH, HF, and SP delivery. The five variables were dichotomized as ‘utilized’ versus ‘not utilized’. Women who attended four or more ANC contacts were categorized as ‘utilized’ for ANC visits. Even though the 2016 WHO ANC model recommends a minimum of eight ANC contacts, the guideline was not yet widely implemented in rural Zambia [29]. Therefore, the previous guideline of four or more ANC visits was used for the analysis. Similarly, if a woman attended all four PNC visits, first within 24 hours of delivery, second within 3 days postpartum, third between 7 and 14 days postpartum, and fourth before 6 weeks postpartum, she was categorized as having utilized PNC visits [30]. If a woman stayed at a MWH at any point of her pregnancy, she was categorized as having a MWH. If a woman delivered her most recent baby at a health post, HF, or a hospital, she was categorized as having utilized a HF and if she delivered with a doctor, clinical officer, nurse, or midwife she was categorized as having delivered with a SP. Each of the RHSs variables were examined individually.

One may argue that utilization of MWHs often increases delivery at HF with SP, and that delivery at HF and delivery with SP are interchangeable. However, because of the limited number of SP, women delivering at a HF does not always lead to delivery with SP [31, 32]. Similarly, in many sub-Saharan African countries, SP travel to women’s homes for delivery in cases of emergency, which means that sometimes women can deliver with a SP without delivering at a HF [32]. Hence, both variables were included as part of the utilization of RHSs.

### Data analysis

To compare the changes in the outcome variables over time between the communities that had access to SILCs and those that did not, interaction effects of the stratified CGs and timepoints (baseline versus endline) were used. This study hypothesized that women from CG3 compared to women from CG1 and women from CG2 will have higher household wealth, higher likelihood to be financially prepared for birth, and higher utilization of RHSs – ANC visits, PNC visits, MWH, HF delivery, and SP delivery – at endline.

Descriptive statistics were analyzed with the means and standard deviation (SD) provided for both the baseline and endline samples as well as the stratified sample between the CGs at baseline and endline. A set of Chi-square tests of independence and independent sample t-tests were implemented to examine the differences in demographic and outcome variables between the baseline and endline participants and participants from the three CGs at baseline and endline.

Interaction effects of CGs and timepoint (i.e., baseline versus endline) were used to assess the relationships between the independent and dependent variables since CGs and timepoint combined have an effect on each of the dependent variables. Linear or logistic regression models without the interaction effect assumes that the effect of each independent variable on the outcome is separate from the other independent variable in the model. Hence, using the interaction effects of CGs and timepoint on outcome variables provides a more accurate understanding of how the inclusion of SILCs in communities influences wealth and maternal health. Key outcome variables were 1) household wealth (wealth index), 2) financial preparedness for birth (saving for most recent delivery), and 3) utilization of RHSs (ANC visits, PNC visits, MWH utilization, HF delivery, and SP delivery). All adjusted models included age, marital status, number of pregnancies, number of live births, and education level. Wealth was also added to the adjusted model when exploring financial preparedness for birth and utilization of RHSs. All analyses accounted for the clustering at the community level by using the vce(cluster) command in Stata. In addition, coefficient (b), standard error (SE), adjusted odds ratios (AORs), and 95% confidence intervals (95%CI) were provided. All statistical analysis was conducted in Stata 17.0 (StataCorp, College Station, TX, USA).

## Results

### Sample demographic characteristics

A total sample of 4711 women were included in the analysis. Approximately half of the sample were from baseline HHS data (*n* = 2381) and the other half from endline HHS data (*n* = 2330). The mean age was 26 years old, and majority were married or cohabiting (87.86%; 86.05%). The average number of pregnancies was 4 at baseline and endline but the average number of live births was 4 at baseline and 3 at endline. Approximately two thirds of the women had some level of primary education and a quarter of the women had secondary education. At baseline, marital status (*p* < 0.001), and education level (*p* < 0.001) were statistically different amongst the three CGs. At endline, marital status (*p* < 0.001), number of pregnancies (*p* = 0.008), number of live births (*p* = 0.005), and education (*p* < 0.001) were statistically different among the three CGs. The comparison of the three CGs at baseline and endline is shown in Table 1.

**Table 1:** Demographic characteristics between Community Groups at baseline and endline

	Baseline					Endline				
	Community Groups					Community Groups				
	Overall	1 = neither MWH nor SILC	2 = only MWH	3 = both MWH and SILC	*p*-value	Overall	1 = neither MWH nor SILC	2 = only MWH	3 = both MWH and SILC	*p*-value
**Total n (%)**	2381 (50.54)	1031 (43.30)	594 (24.95)	756 (31.75)		2330 (49.46)	1113 (47.77)	619 (26.57)	598 (25.67)	
**Age**					0.716					0.379
Mean (SD)	26.11 (6.96)	26.22 (7.11)	25.93 (6.71)	26.09 (6.97)		26.08 (6.94)	25.97 (6.92)	26.35(7.03)	26.01 (6.88)	
**Marital Status n (%)**					< 0.001***^a,b^					0.001***^a,b^
Married/Cohabiting	2092 (87.86)	890 (86.32)	511 (86.03)	691 (91.40)		2005 (86.05)	946 (85.00)	522 (84.33)	537 (89.80)	
Divorced/Separated/Widowed	125 (5.25)	53 (5.14)	29 (4.88)	43 (5.69)		118 (5.06)	62 (5.57)	31 (5.01)	25 (4.18)	
Single	159 (6.68)	86 (8.34)	52 (8.75)	21 (2.78)		180 (7.73)	95 (8.54)	63 (10.18)	22 (3.68)	
**Number of pregnancies**					0.400					0.008**^b^
Mean (SD)	3.86 (2.54)	3.95 (2.61)	3.85 (2.44)	3.75 (2.51)		3.75(2.42)	3.74 (2.46)	3.94 (2.41)	3.57 (2.33)	
**Number of live births**					0.068 ^a^					0.005** ^b^
Mean (SD)	3.59 (2.35)	3.68 (2.43)	3.64 (2.28)	3.42 (2.28)		3.38 (2.39)	3.39 (2.38)	3.55 (2.46)	3.16 (2.33)	
**Education n (%)**					< 0.001*** ^a, b^					< 0.001*** ^a, b^
None	362 (15.20)	160 (15.52)	83 (13.97)	119 (15.74)		280 (12.02)	152 (13.66)	63 (10.18)	65 (10.87)	
Primary	1444 (60.65)	603 (58.49)	332 (55.89)	509 (67.33)		1370 (58.80)	628 (56.42)	350 (56.54)	392 (65.55)	
Secondary	568 (23.86)	266 (25.80)	177 (29.80)	125 (16.53)		650 (27.90)	323 (29.02)	203 (32.79)	124 (20.74)	

Independent sample t-test and Pearson chi-square test performed for categorical variables; The percentage for each of the variables reflects missing data

**p < 0.01; ***p < 0.001

^a^ statistical significance between community group 1 (neither MWH nor SILC) and community group 3(both MWH & SILC)

^b^ statistical significance between community group 2 (only MWH) and community group 3 (both MWH & SILC)

Descriptive statistics for the outcome variables between CGs and timepoint are provided in Table 2. At baseline, women among CG1 were generally evenly distributed between the wealth quintiles, while the highest percentage of women among CG2 belonged to second richest group (25.25%), and the highest percentage of women among CG3 belonged to the poorest group (22.75%). At endline, the highest percentage of women among CG1 belonged in the poorest group (18.6%), the highest percentage of women among CG2 remained in the second richest group (23.59%), and the highest percentage of women among CG3 also remained in the poorest group (27.76%). At baseline, 82% of all women saved for their most recent delivery, 58% of the women attended four or more ANC visits (58%), and 53% of the women did not attend any PNC visits. At endline, 75% of the women saved for most recent delivery, 71% of the women attended four or more ANC visits, and 41% of the women did not attend any PNC visits. Finally, at baseline, 31% of the women stayed at a MWH, 81% delivered at a HF, and 56% of the women delivered with a SP. At endline, 35% of the women stayed at a MWH, 89% delivered at a HF, and 84% of the women delivered with a SP. The percentages for all the variables in Tables 1 and 2 reflect missing observation with wealth index (baseline: 351; 14.71%; endline: 299; 12.83%) and most recent delivery by skilled provider (baseline: 562;23.6%; endline: 55; 2.36%) having the largest missing observations.

**Table 2:** Descriptive statistics for outcome variables between Community Groups at baseline and endline

	Baseline					Endline				
	Community Groups					Community Groups				
	Overall	1 = neither MWH nor SILC	2 = only MWH	3 = both MWH and SILC	*p*-value	Overall	1 = neither MWH nor SILC	2 = only MWH	3 = both MWH and SILC	*p*-value
**Total n (%)**	2381 (50.54)	1031 (43.30)	594 (24.95)	756 (31.75)		2330 (49.46)	1113 (47.77)	619 (26.57)	598 (25.67)	
**Wealth index n (%)**					< 0.001***^a,b,c^					< 0.001***^a,b, c^
Poorest	402 (16.88)	182 (17.65)	48 (8.08)	172 (22.75)		411 (17.64)	207 (18.60)	38 (6.14)	166 (27.76)	
Poor	438 (18.40)	189 (18.33)	88 (14.81)	161 (21.30)		374 (16.05)	167 (15.00)	85 (13.73)	122 (20.40)	
Middle	405 (17.01)	166 (16.10)	111 (18.69)	128 (16.93)		407 (17.47)	194 (17.43)	104 (16.80)	109 (18.23)	
Rich	409 (17.18)	172 (16.68)	150 (25.25)	87 (11.51)		403 (17.30)	183 (16.44)	146 (23.59)	74 (12.37)	
Richest	376 (15.79)	191 (18.53)	127 (21.38)	58 (7.67)		436 (18.7)	219 (19.68)	166 (26.82)	51 (8.53)	
**Saved for most recent delivery n (%)**					0.504					0.063 ^a^
No	412 (17.30)	180 (17.46)	110 (18.52)	122 (16.14)		549 (23.56)	276 (24.80)	153 (24.72)	120 (20.07)	
Yes	1957 (82.19)	847 (82.15)	480 (80.81)	630 (83.33)		1768 (75.88)	828 (74.39)	465 (75.12)	475 (79.43)	
**ANC**					0.210					0.152
Less than four times	982 (41.24)	406 (39.38)	257 (43.27)	319 (42.20)		666 (28.58)	313 (28.12)	165 (26.66)	188 (31.44)	
Four or more times	1392 (58.46)	625 (60.62)	334 (56.23)	433 (57.28)		1660 (71.24)	798 (71.70)	454 (73.34)	408 (68.23)	
**PNC**					< 0.001*** ^a, b, c^					0.001*** ^a, c^
None	1285 (53.97)	576 (55.87)	370 (62.29)	339 (44.84)		971 (41.67)	525 (47.17)	236 (38.13)	210 (35.12)	
Less than four times	945 (39.69)	398 (38.60)	198 (33.33)	349 (46.16)		1054 (45.24)	468 (42.05)	308 (49.76)	278 (46.49)	
All four times	149 (6.26)	56 (5.43)	26 (4.38)	67 (8.86)		211 (9.06)	78 (7.01)	64 (10.34)	69 (11.54)	
**Stayed at MWH**					0.037* ^b, c^					0.001*** ^a, c^
No	1622 (68.12)	712 (69.06)	380 (63.97)	530 (70.11)		1193 (51.20)	710 (63.79)	256 (41.36)	227 (37.96)	
Yes	747 (31.37)	314 (30.46)	211 (35.52)	222 (29.37)		1130 (48.50)	399 (35.85)	363 (58.64)	368 (61.54)	
**Most recent delivery location**					0.012* ^b, c^					0.001*** ^a, b^
Your home/Other home/On the road/Other	445 (18.69)	188 (18.23)	134 (22.56)	123 (16.27)		241 (10.34)	134 (12.04)	72 (11.63)	35 (5.85)	
Health post/Facility/Hospital	1931 (81.10)	843 (81.77)	459 (77.27)	629 (83.20)		2089 (89.66)	979 (87.96)	547 (88.37)	563 (94.15)	
**Most recent delivery by skilled care provider**					0.727					0.001*** ^a, b^
No	481 (20.20)	203 (19.69)	113 (19.02)	165 (21.83)		302 (12.96)	169 (15.18)	88 (14.22)	45 (7.53)	
Yes	1338 (56.19)	564 (54.70)	336 (56.57)	438 (57.94)		1973 (84.68)	915 (82.21)	526 (84.98)	532 (88.96)	

Independent sample t-test and Pearson chi-square test performed for categorical variables; The percentage for each of the variables reflects missing data

*p < 0.05; ***p < 0.001

^a^ statistical significance between Group 1 of communities (neither MWH nor SILC) and Group 3 (both MWH & SILC)

^b^ statistical significance between Group 2 of communities (only MWH) and Group 3 (both MWH & SILC)

^c^ statistical significance between Group 1 of communities (neither MWH nor SILC) and Group 2 of communities (only MWH)

There were significant differences between the CGs at baseline for household wealth (*p* < 0.001), PNC visits (*p* < 0.000), MWH utilization (*p* = 0.037), and HF delivery (*p* = 0.012). Furthermore, there were significant differences between the CGs at endline for household wealth (*p* < 0.001), PNC visits (*p* < 0.001), MWH utilization (*p* < 0.001), HF delivery (*p* < 0.001), and delivery with a SP (*p* < 0.001). Missing data from each variable in both Tables 1 and 2 were accounted for in the percentage.

### Household wealth and financial preparedness for birth

Table 3 shows there is no interaction effect between CGs and timepoint on household wealth and financial preparedness for birth.

**Table 3:** Interaction effect of community groups and timepoint on wealth and saving for most recent delivery

	Wealth index	Saved for most recent delivery
**Community groups**	Adjusted b (SE){95% CI}	AOR (95% CI)
1 = neither MWH nor SILC	0.39 (0.14) {0.09–0.68} *	0.72 (0.45–1.17)
2 = only MWH	0.73 (0.08) {0.55–0.91} ***	0.64 (0.40–1.01)
3 = both MWH and SILC	Ref	Ref
**Time point**		
Baseline	Ref	Ref
Endline	−0.07 (0.07) {−0.22–0.07}	0.71 (0.42–1.18)
**Community group X time point**^**a**^		
1 = neither MWH nor SILC X End Line	0.11 (0.10) {−0.09–0.32}	0.88 (0.47–1.65)
2 = only MWH X End Line	0.18 (0.10) {−0.02–0.40}	0.98 (0.52–1.84)
3 = both MWH and SILC X End line	Ref	Ref

All adjusted logistic and linear regression models controlled for age, marital status, gravida, parity, education, community group, and timepoint. Please refer to Table 1 for more details on these variables

All analysis were clustered at the community level

*AOR* Adjusted odds ratio, *CI* Confidence interval, *b* Unstandardized coefficient, *SE* Standard error, *MWH* Maternity waiting homes, *SILC* Savings and internal lending communities

*p < 0.05; ***p < 0.001; ^a^ z test for equality comparing CG1 and CG2 showed insignificant results for wealth index and saved for most recent delivery

### Utilization of RHSs

Findings reported in Tables 4 and 5 show the interaction effect of CGs, timepoint, and utilization of RHSs. Table 4 shows that CGs and timepoint did not have a significant interaction effect on attending four or more ANC visits and attending all four PNC visits. Table 5, however, shows the interaction effect of CGs and timepoint on MWH utilization, HF delivery, and SP delivery. Women from CG1, with neither MWHs nor SILCs, at endline had 0.65 times lower odds (95%CI: 0.18–0.71) of utilizing MWHs than women from CG3, with both MWHs and SILCs. Furthermore, women from CG1 at endline had 0.5 times lower odds of delivering at a HF (95%CI: 0.32–0.78) compared to women from CG3. Additionally, women from CG 1(AOR: 0.34; 95%CI: 0.17–0.66) and CG2 (AOR: 0.33; 95%CI: 0.17–0.64) had lower odds of delivering with a SP [33].

**Table 4:** Interaction effect of community groups and timepoint on antenatal care visit and postnatal care visits

	**>** 4 or more ANC visits	All 4 PNC visits
**Community groups**	AOR (95% CI)	AOR (95% CI)
1 = neither MWH nor SILC	1.09 (0.76–1.55)	0.96 (0.50–1.88)
2 = only MWH	0.86 (0.59–1.24)	0.87 (0.42–1.80)
3 = both MWH and SILC	Ref	Ref
**Time point**		
Baseline	Ref	Ref
Endline	1.43 (0.88–2.33)	2.60 (1.47–4.58) **
**Community group X time point**^**a**^		
1 = neither MWH nor SILC X End Line	1.13 (0.68–1.88)	0.58 (0.22–1.50)
2 = only MWH X End Line	1.46 (0.80–2.67)	1.04 (0.45–2.36)
3 = both MWH and SILC X End line	Ref	Ref

All adjusted logistic regression models controlled for age, marital status, gravida, parity, education, wealth (quintiles), community group, and timepoint. Please refer to Table 1 for more details on these variables. All analysis were clustered at the community level

*AOR* Adjusted odds ratio, *CI* Confidence interval, *MWH* Maternity waiting homes, *SILC* Savings and internal lending communities, *ANC* Antenatal care, *PNC* Postnatal care

**p < 0.01; ^a^ z test for equality comparing CG1 and CG2 showed insignificant results for four or more ANC visits and all 4 PNC visits

**Table 5:** Interaction effect of community groups and timepoint on utilization of maternity waiting homes, delivery at a health facility, and delivery with skilled provider

	Utilization of MWHs	Most recent delivery at HF	Most recent delivery with SP
**Community group**	AOR (95% CI)	AOR (95% CI)	AOR (95% CI)
1 = neither MWH nor SILC	1.03 (0.48–2.19)	0.84 (0.47–1.49)	1.03 (0.58–1.83)
2 = only MWH	1.26 (0.50–3.12)	0.61 (0.28–1.32)	1.02 (0.51–2.02)
3 = both MWH and SILC	Ref	Ref	Ref
**Time point**			
Baseline	Ref	Ref	Ref
Endline	3.35 (1.92–5.85)	3.35 (2.39–4.69) ***	5.75 (3.32–9.95) ***
**Community group X time point**^**a**^			
1 = neither MWH nor SILC X End Line	0.35 (0.18–0.71) **	0.50 (0.32–0.78) **	0.34 (0.17–0.66) **
2 = only MWH X End Line	0.65 (0.32–1.31)	0.64 (0.39–1.04)	0.33 (0.17–0.64) **
3 = both MWH and SILC X End line	Ref	Ref	Ref

All adjusted logistic regression models controlled for age, marital status, gravida, parity, education, wealth (quintiles), community group, and timepoint. Please refer to Table 1 for more details on these variables. All analysis were clustered at the community level

*AOR* Adjusted odds ratio, *CI* Confidence interval, *MWH* Maternity waiting homes, *SILC* Savings and internal lending communities, *HF* Health facilities, *SP* Skilled provider

**p < 0.01; ***p < 0.001; ^a^ z test for equality comparing CG1 and CG2 showed insignificant results for utilization of MWHs, most recent delivery at HF, but significant for most recent delivery with SP (z: − 2.18)

In summary, statistically significant interaction effects of CGs and timepoint were only observed for MWH utilization, HF delivery, and SP delivery. The odds of utilizing MWHs and delivering at a HF were significantly lower for women from communities with neither MWHs nor SILCs compared to women from communities with both MWHs and SILCs at endline. However, regarding delivery with SP, both women from communities with neither MWHs nor SILCs and women from communities with only MWHs had lower odds compared to women from communities with both MWHs and SILCs at endline. CGs and timepoint together had no effect on household wealth, financial preparedness for birth, attending four or more ANC visits, and attending all four PNC visits.

## Discussion

In terms of household wealth, the results showed that CG and timepoint together had no significant association with household wealth. This finding does not support our hypothesis that women from communities with SILCs would have been able to accumulate more household wealth. However, the result adds to the ongoing debate regarding the economic impact of SGs [34]. A three-year randomized control trial examining the impact of SGs in Mali found no change in income and health expenditures, with marginally significant increase in education expenditures and livestock holdings [34]. A cluster randomized evaluation study conducted in Ghana, Malawi, and Uganda concluded that SGs lead to improvement in household business outcomes but no impact on average consumption or other livelihoods [35].

One explanation for the results showing no statistically significant association between access to SILCs and household wealth may be due to the measure used to capture wealth. Using household assets and quality of housing and water supply is a valid and commonly used proxy for economic status [36]. We argue that women from CGs with SILCs may have used the savings and loans from SILCs to purchase other household assets and/or invest in areas such as education and food that may not have been captured in the HHS data. These purchases and improvements are often mentioned when SG participants usage of funds are analyzed [35, 37].

Another possible explanation may be related to the implementation period. The SILCs were first implemented in early 2016, and the endline data were collected in August and September of 2018. Two and a half years of implementation does not appear to be short considering many SG implementation periods generally range from one to three years [34, 35]. However, some experts argue that this is not sufficient time to examine the significance of financial effects that can result from participating in a SG [13]. For example, a randomized control trial conducted in Mali over 3 years suggested that the study may have been too short to capture any changes produced by savings cycles [34]. Considering that women from CG3 with both MWHs and SILCs were the poorest of the three CGs at both baseline and endline, this may suggest that the economic benefit of SILCs had not yet been produced within the two and half year timespan.

In summary, the results show there is no significant association between access to SILCs and household wealth, adding to the mixed results in the literature regarding the economic impact of SGs. The results should be interpreted cautiously considering the limitation in the measure of household wealth and the potentially short implementation period.

In terms of financial preparedness for birth, the analysis found that the interaction between CGs and timepoint together had no effect on financial preparedness for birth. While SILC participation may have allowed participants to better understand and prioritize financial resources for birth, it may not have led to enough increase in wealth to save for the most recent delivery at endline. SGs such as SILCs, have however, been shown to be a conducive platform for participants to discuss personal and communal joys and difficulties, including pregnancy and childbirth [13, 16]. Such communal discussions and sharing have shown to increase understanding and knowledge with behavioral implications such as an increase in facility delivery [13]. However, the lack of a significant increase in household wealth may contribute to the limited amount of money to save for birth.

In terms of utilization of RHSs, the interaction between CGs and timepoint was statistically significant for utilizing MWHs, delivering at a HF, and delivering with a SP. One potential explanation for the lack of a statistically significant association between CGs and timepoint for ANC and PNC visits may be due to the conservative measure of the two variables. Per WHO guidelines during the implementation period, ANC was captured as women attending four or more ANC visits, and PNC as attending all four PNC visits [29, 30]. For the survey to have captured women’s utilization of ANC and PNC visits, women had to travel to the HF multiple times, potentially requiring multiple out of pocket costs and opportunity costs. A recent systematic review examining the cost of various RHSs in LMICs found the average cost per service, excluding transportation costs and productivity loss ranged between US$7.24–$31.42 for ANC and US$5.04 for PNC [38]. Considering that the communities included in the present study are predominantly rural and far from the nearest HFs, recurring expenses such as transportation and the loss of productivity for each ANC and PNC visit may have deterred women from prioritizing their financial resources to attend all of the required ANC and PNC visits [4].

With standardized high-quality MWHs implemented by the parent study, it is not surprising that communities with access to MWHs had higher likelihood of MWH utilization and delivery at a HF. However, women from communities with neither MWHs nor SILCs and women from communities with only MWHs had lower odds of delivering with a SP. This study suggests that even when women stayed at a MWH and delivered at a HF, she may not have delivered with a skilled provider. This highlights the importance of healthcare quality, including skilled healthcare providers being present to provide care. By providing a dwelling place near the HF for pregnant women, MWHs aim to address the second delay, the delay in reaching care, in Thaddeus and Maine’s three delay model [39, 40]. However, if the HF is unable to deliver quality healthcare –including health care providers, medication, and equipment being readily available – the third delay, delay in receiving care, remains a barrier to safe pregnancy and childbirth. Future studies need to investigate the gaps between the second and third delay, reaching and receiving care, and means to improve the continuity of care to ultimately improve maternal health.

Another potential explanation of women from communities with MWHs and SILCs having higher odds of accessing MWHs, as well as delivering at a HF with a SP may be due to the community’s increased social capital. Social capital is often defined as dense networks of social interaction that may emerge through a person’s networks and participation in community events [41]. Such networks lead to a wide range of shared awareness, knowledge, and information that can have tangible effects such as increased contraceptive use and increased child survival [42]. It is well-established how SGs can increase participants’ social capital to ultimately influence their health and their family’s health [14]. Similarly, with the increased opportunities to share about pregnancy and childbirth experiences and resources, communities with SILCs may have increased knowledge and awareness regarding the importance of HF delivery and delivery with a SP.

While wealth assessed using household assets and housing quality may not have increased significantly, SILCs may still have allowed women to set aside financial resources for HF delivery and delivery with a SP. Of the costs related to various RHSs, costs related to delivery are often the highest, ranging from US$14.3 to $378.94 in LMICs depending on the facility type, provider type, and complexity of care [38]. A study conducted in rural Zambia showed the average out-of-pocket cost for delivery was US$28.76, approximately one third of the monthly household income of the poorest Zambian households [8]. Therefore, when financial resources are scant and women are not able to access the full continuum of RHSs combined with the increased collective awareness regarding the importance of HF delivery and delivery with a SP, women from communities with both MWH and SILCs may have prioritized their resources for delivery related expenses.

### Limitations

This study has several limitations. First, because different forms of SGs are prevalent throughout rural Zambia, it is subject to contamination. Considering that World Vision alone has implemented approximately 25,000 SGs across Zambia, it is possible that there were SGs in CG1 (no MWH or SILC) and CG2 (MWH only) [43]. Second, the three CGs had significantly different baseline characteristics that may have influenced the results. However, the interaction terms were used to control for the time variant differences in the outcome variables. Additionally, all the statistical models control for these different characteristics at baseline. Third, the baseline HHS data were collected April and May of 2016, a few months after the SILCs were first introduced in the communities in January 2016. However, the impact of SGs is often assessed after at least one full cycle, usually ranging from ten to twelve months of SILC participation. Therefore, a few months of SILC participation may not have had a significant effect when baseline data were collected. Fourth, the stratified CGs do not include a SILCs only group. Therefore, it identifies the effect of having access to SILCs not by comparing the communities with only SILCs to the control group but by comparing the communities with only MWHs and those with both MWHs and SILCs, Lastly, the HHS did not capture the true number of survey participants from different communities who actually participated in the SILCs. Therefore, the results should be interpreted as having access to SILCs, not participating in them.

## Conclusion

The present study aimed to understand the association between having access to SILCs and: 1) household wealth, 2) financial preparedness for birth, and 3) utilization of RHSs. This study found that CG and timepoint together did not lead to a significant increase in household wealth, saving for the most recent delivery, utilization of four or more ANC visits, or attending all four PNC visits. This may be due to the short implementation period that was not enough to lead to drastic change in household wealth.

Regarding utilization of MWHs, HF delivery, and SP delivery, CGs with neither MWHs nor SILCs had significantly lower utilization of MWHs, HF delivery, and SP delivery compared to communities with both MWHs and SILCs at endline. This result may be due to healthcare provider absenteeism, increased social capital of communities with access to SILCs, and/or increased sharing of knowledge and information stemming from a stronger sense of community and trust. With increased knowledge and awareness but limited financial resources, women from communities with access to SILCs may have chosen to prioritize resource for delivery rather than ANC and PNC. More effort needs to be dedicated to understanding and empowering poor women living in rural areas to access the full continuum of RHSs. Furthermore, health facilities also should be strengthened to provide quality health care. In sum, the present study holds crucial implications regarding the economic potential of SILCs to help women of LMICs to access fundamental RHSs to promote both their and their children’s health.

## Data Availability

The data that support the findings of this study are available from the corresponding author upon reasonable request.

## Abbreviations

RHS: Reproductive Health Service
ANC: Antenatal Care
PNC: Postnatal Care
MWH: Maternity Waiting Home
HF: Health Facility
SP: Skilled Provider
LMIC: Low- and Middle-Income Countries
SG: Savings Groups
SILC: Savings and Internal Lending Communities
MHA: Maternity Home Alliance
HHS: Household Survey
CG: Community Group
